# Soil microbiomes as sources and sinks of greenhouse gases. Challenges and opportunities

**DOI:** 10.1093/sumbio/qvaf033

**Published:** 2025-12-13

**Authors:** Caroline Hayley Orr, Austine Iyalekhue Otabor, Odunayo Olabimtan, Taiye Onaivi, David Opoku, Saadlee Shehreen

**Affiliations:** School of Health and Life Sciences, Teesside University, Middlesbrough, TS1 3BX, United Kingdom; National Horizons Centre, Teesside University, Darlington, DL1 1HG, United Kingdom; School of Health and Life Sciences, Teesside University, Middlesbrough, TS1 3BX, United Kingdom; School of Health and Life Sciences, Teesside University, Middlesbrough, TS1 3BX, United Kingdom; School of Health and Life Sciences, Teesside University, Middlesbrough, TS1 3BX, United Kingdom; School of Health and Life Sciences, Teesside University, Middlesbrough, TS1 3BX, United Kingdom; School of Health and Life Sciences, Teesside University, Middlesbrough, TS1 3BX, United Kingdom; National Horizons Centre, Teesside University, Darlington, DL1 1HG, United Kingdom

## Abstract

Biogeochemical cycles, including the microbially mediated carbon and nitrogen cycles, within soils govern the health status of environments, and are impacted, and impact upon, climate change. Anthropogenic activities have shifted the amounts and forms of carbon and nitrogen within soils. Whilst access to carbon and nitrogen is essential for plant growth and microbial food webs, excess carbon and nitrogen can lead to pollution via leaching into groundwater and the release of greenhouse gases, notably carbon dioxide, methane and nitrous oxide, contributing to global warming. In addition, changes in environmental conditions brought about by climate change, such as temperature increases, increased acidification of environments and shifts in soil moisture can also impact on microbial communities residing within soils leading again to shifts in biogeochemical cycling. Therefore, understanding the microbial communities associated with biogeochemical cycling is key to understanding healthy environments. This review summarises the known roles of the microbial community within biogeochemical cycling and the impacts on these communities from climate change. We also explore the additional opportunities for understanding impacts of climate change and future practices for sustainable agriculture via utilising soil bacteriophage within monitoring and their potential as biocontrol agents.

**Sustainability Statement**

This article is associated with UN Sustainable Development Goal SDG 13 (Climate Action) with relevance to SDG 15 (Life on Land) and SDG 12 (responsible consumption and production). Globally there is a need to increase food availablity by improving agricultural practices. This drives intensification of farming which has negative impacts on biogeochemical cycling within soils, resulting in release of gaseous emissions and leaching of pollutants. Understanding soil biogeochemical cycling, and developing better monitoring and biocontrol agents, potentially through the use of bacteriophage can ultimately lead to more sustainable farming which will be more robust in the face of changing climate.

## Impact of climate change on soils and soil microbiomes

Climate change is an immediate threat to all environments and remains one of the most pressing environmental challenges of the 21st century (Buhaug et al. 2023). The Sixth Assessment Report of the Intergovernmental Panel on Climate Change (IPCC) projects that global temperatures are more than 50% likely to exceed the 1.5°C threshold, potentially well before mid-century under high-emissions scenarios (McKay et al. 2022). Central to this warming trend is the accumulation of greenhouse gases (GHGs) such as methane (CH_4_), carbon dioxide (CO_2_), and nitrous oxide (N_2_O), which trap heat in the atmosphere and intensify global warming (Filonchyk et al. 2024, Bolan et al. 2024). Additionally, climate changes lead to changes in precipitation events resulting in flooding and soil erosion which in turn also impact on soil biogeochemical cycles (Eekhout and de Vente 2022).

This review summarises how climate change impacts on microbially mediated biogeochemical cycling within soils. Soils are a critical ecosystem which support plant life, regulate water, cycle nutrients and store carbon. A recent review by Kopittke (et al. 2023) et al. 2023) highlighted the five main functions of soil in providing a healthy environment for plant growth and overall ecosystem health as: a provider of biomass via agricultural systems, a regulator of dynamic carbon stores, a habitat for 25% of global biodiversity, a key location for nutrient cycling and a major contributor to water cycling.

Many of the processes occurring within soils are mediated by the complex communities of bacteria, fungi, archaea and other microorganisms which reside there. Soils are home to most of the terrestrial biodiversity with estimates of up to 1 billion bacterial cells, 200 m of fungal hyphae and trillions of viruses all of which perform essential roles within our environments and many of which are involved in biogeochemical cycling (Bardgett and van der Putten 2014, Fierer 2017, Xiong and Lu 2022).

Climate change by its nature can affect soil properties such as moisture—with increased periods of wetting and drying, temperature, pH—with the dissolution of carbon dioxide leading to soil acidification, and organic matter content, via impacts on soil organic carbon. Each of these are key drivers of microbial diversity change (as reviewed by Philippot et al. 2024) meaning shifts within soils caused by climate change could lead to changes in microbial community structure and function which can impact the ability of soils to perform successfully. As discussed later, changes in microbial activity associated with carbon and nitrogen cycling can influence greenhouse gas emissions (e.g. CO_2_, CH_4_, N_2_O), potentially worsening the situation.

Any shift in microbial community structure can have an impact on key ecosystem functioning such as plant-microbe interactions, soil health and agricultural productivity. The impact of climate change on microbial communities is complex and sometimes contradictory. While some studies report increased microbial biomass under warming and drought (Jiang et al. 2020), others show a reduction in microbial consumers and diversity (Basińska et al. 2020). These structural changes can destabilise carbon balance, enhancing microbial respiration and methane emissions (Hopple et al. 2020). Yet, other research finds minimal or no impact, suggesting that microbial responses depend on the magnitude and duration of climate change, as well as experimental design (Briske et al. 2006, Jassey et al. 2015, Lamit et al. 2021, Xue et al. 2021).

To resolve these uncertainties, further research is needed into changes in microbial alpha and beta diversity, abundance, and functional traits under climate stress. Experimental studies have shown that rising air temperatures may increase CO_2_ fixation while reducing respiration, potentially offsetting some carbon losses (Le Geay et al. 2024). Understanding these compensatory mechanisms is essential for predicting long-term environmental responses to climate change.

Here, we consider some of the emerging tools such as metagenomics and other omics technologies which can be used in uncovering microbial responses to climate change, as well as considering interesting future lines of enquiry for research in determining climate-resilient soil management practices.

## Climate change impacts on the global carbon cycle and associated soil microbiomes

The carbon cycle is a vital biogeochemical process that connects all life forms on earth and bridges biotic and abiotic components of ecosystems. Climate change stands out as one of the most urgent challenges of the 21^st^ century, demanding innovative solutions across science, policy, and society (Beattie et al. 2024). Given that the carbon cycle is heavily influenced by temperature-sensitive processes, the result is an intricate climate-carbon cycle coupling (Willeit et al. 2014). The numerous ways in which climate change has impacted and will continue to impact the carbon cycle have been well documented in literature (Friedlingstein et al. 2022).

Advancements in Earth System Models (ESMs) have enabled scientists to simulate how the climate system may respond to anthropogenic greenhouse gas emissions throughout the 21^st^ Century and beyond (Jones et al. 2016). ESMs and their predecessors (traditional land or ocean carbon cycle models) were designed to answer two foundational questions. First, is observed climate change attributable to human activity or is it a natural Earth System curve? Secondly, how will the climate system including land and ocean sinks will respond to different future emissions scenarios (Friedlingstein 2015). The first question is no longer a debate in the scientific community following overwhelming evidence that human activities have unequivocally warmed the atmosphere, ocean and land (Jungclaus et al. 2018, Allan et al. 2023). The second question is more complex due to the presence of feedbacks in the climate system. (Friedlingstein and Prentice 2010, Willeit et al. 2014, Arora et al. 2020).

Climate feedbacks, also called biosphere feedbacks are generally classified as either biogeochemical or biogeophysical processes that amplify (positive feedbacks) or dampen (negative feedbacks) the effects of climate change (Prentice and Williams 2015). A breakdown of fast physical climate feedbacks and Earth System feedbacks is provided in Heinze et al. (2019). Modelling studies generally report a net positive climate feedback on the carbon cycle where warming accelerates carbon release from natural reservoirs (Friedlingstein et al. 2003, Scheffer et al. 2006, Matthews and Keith 2007). For example, (Melillo et al. 2002) estimated carbon losses of 944 g m^−2^ with warming over a 10-year period, the equivalent of 11.3% of the soil carbon found in the top 60 cm of the soil profile. Nonetheless, some studies have also reported negative feedbacks (Torres Mendonça et al. 2024).

The fate of carbon sinks, both land and ocean, will depend on several interconnecting factors such as timescales, feedback intensity and future emissions scenarios (Chowdhury 2013). While there have been divergent modelling results and interpretations surrounding the long-term efficiency of carbon sinks under different future emissions scenarios and climate feedbacks (Gloor et al. 2010, Schwinger et al. 2014, Mystakidis et al. 2016, Zhang et al. 2021), the analyses tend to be clearer when they effectively incorporate timescales. Notably, over shorter timescales, studies show that the land or terrestrial carbon cycle dominates atmospheric carbon dioxide dynamics, while the marine carbon cycle will profoundly take up more carbon over longer periods (Willeit et al. 2014, Friedlingstein 2015). Terrestrial ecosystems, in particular, are the primary drivers of the seasonal carbon dioxide cycle (Graven et al. 2013), making them critical for the development of climate change mitigation strategies. As a result, there is a substantial interest in understanding the biological pathways which govern the terrestrial carbon cycle and how these pathways might be influenced by climate change.

### Carbon dioxide

The respiratory flux of carbon dioxide from soils, driven primarily by microbial metabolism, dwarfs emissions from fossil fuel combustion because soils constitute the main carbon stocks of terrestrial ecosystems (Bardgett et al. 2008). Even small perturbations in soil microbial activity can lead to significant shifts in the atmospheric concentration of carbon dioxide (Johnston et al. 2004, Gougoulias et al. 2014). The work of Friedlingstein et al. (2003) underscored that land-based climate feedbacks were fundamentally responsible for large discrepancies in carbon cycle model outputs. These findings highlight the importance of explicitly incorporating microbial biomes or their functional diversity in Earth System Models in a bid to maximize our understanding of the so-called climate-microbe feedbacks and their influence on land-based carbon dioxide dynamics.

Climate-microbe feedbacks can be categorised into direct and indirect feedbacks. In direct feedbacks, climate variables directly affect microbial physiology, community structure or metabolism. Indirect feedbacks are based on climate-driven changes in non-microbial factors (e.g. vegetation changes, litter inputs, soil moisture) that modify microbial communities and function (Bardgett et al. 2008). Warming is perhaps the most widely cited example of direct feedbacks and increase in carbon dioxide efflux to the atmosphere from accelerated heterotrophic microbial respiration is the most frequently reported microbial response (Davidson and Janssens 2006, Chen et al. 2024). Other examples of warming-induced microbial responses reported in literature include physiological activation (Metze et al. 2024), metabolic shifts (DeAngelis et al. 2015) and community restructuring (Salazar et al. 2020). Indirect feedbacks, due to plant community structures may also modulate the direct effects of climate variables on microbial communities and structures. For example, Fahey and colleagues showed that changes in plant richness and root biomass interactively shaped soil fungal and bacterial diversity and functional groups (Fahey et al. 2020). In some cases, indirect feedbacks may be more dominantly responsible for shifts in microbial enzyme activity and composition when combined with direct feedbacks (Deltedesco et al. 2020). Whether direct or indirect, the evidence suggests that climate change can reorganise microbial community structure and function, potentially altering the balance between carbon storage and release. The outcome could be a net positive or negative climate-microbe feedback with ramifications for the climate-carbon cycle system.

Understanding the impacts of climate-microbe feedbacks on the terrestrial carbon cycle and how the knowledge can be used for climate change mitigation, represents a significant research gap requiring transdisciplinary partnerships and new technological frontiers (Gougoulias et al. 2014, Lennon et al. 2024). Despite an overall lack of climate-microbe representation in most ESMs (Lennon et al. 2024), some progress has been recorded with great potential for further improvements. For example, in the MIcrobial-MIneral Carbon Stabilization (MIMICS) model, findings showed that soil carbon accumulation rates were slower in response to elevated atmospheric carbon dioxide concentration than reported in conventional ESMs (Wieder et al. 2015). Another study simulating soil carbon variations using the CLM-Microbe model by He et al. (2021) demonstrated that model performance is enhanced when microbial mechanisms are explicitly represented. An outline of top ten priorities, opportunities and challenges for integrating microorganisms into ESMs for climate change prediction is documented in Lennon et al. (2024).

We can infer that soil microbiome-mediated belowground storage of carbon provides an opportunity to manipulate components of the terrestrial biosphere to sequester carbon and reduce atmospheric emissions. The aims of such experiments are to slow decomposition and stabilize soil organic matter and enhance microbial pathways that promote long-term storage. Perhaps, the first step towards such efforts will be to phylogenetically identifying genes for microbial carbon use efficiency (CUE), the proportion of substrate carbon converted into microbial biomass versus lost as carbon dioxide, in order to identify beneficial soil microorganisms (Gougoulias et al. 2014). Bardgett et al. (2013) proposed a generic framework that stratifies microbial response to climate perturbations based on individual responses, community reordering and species immigration and loss. They suggested that the climate-microbe interactions vary across several scales from local individual responses to more persisting large-scale impacts due to community restructuring and immigration. Knowledge of these interactions may be used to conduct soil microbiome manipulative experiments designed to limit greenhouse gas emissions and increase carbon stocks especially in managed soils. For example, Beattie et al., (2024) argued that such microbial interventions can help to boost soil carbon stocks directly (e.g. through microbial strains and soil transplant) or indirectly (e.g. isolation of microbial-catalysed processes).

### Methane

In addition to focus on release of CO_2_, attention should also be given to release of methane (CH_4_) from soil environments. Whilst this is an issue for agricultural systems it is particularly pertinent within peatlands. Peatlands are among the most efficient terrestrial ecosystems for long-term carbon storage, owing to their waterlogged, anaerobic conditions that slow organic matter decomposition. However, peatland degradation significantly alters their carbon dynamics, with profound implications for greenhouse gas (GHG) emissions (Antala et al. 2022). Among these gases, methane is particularly important due to its high global warming potential which far exceeds that of carbon dioxide, especially upon initial release (Abdalla et al. 2016).

Methane concentrations in the atmosphere have risen sharply, driven by anthropogenic activities such as fossil fuel extraction, biomass burning, waste management, and wetland alteration (Whalen 2005, Savi et al. 2016). Wetlands, including peatlands, are natural sources of methane, primarily through the activity of methanogenic archaea in anoxic soil layers. These microorganisms produce methane as a metabolic byproduct, while methanotrophic bacteria in the aerobic zones oxidize methane to CO₂, acting as a biological filter (Le Mer and Roger 2001).

Methane production in peatlands depends on substrates such as methyl sulfides, methylamines, formate, acetate, methanol, and CO₂/H₂—byproducts of organic matter decomposition by fungi and bacteria. Under anaerobic conditions, the enzyme methyl-coenzyme M reductase catalyses the final step of methanogenesis, releasing methane and ATP for microbial metabolism (Gendron and Allen 2022).

Peatlands, formed through climatic influences on wetlands, cover approximately one-third of global soil and are critical to understanding biospheric responses to climate change (Tveit et al. 2012). Their acidic, low-redox, and carbon-rich soils inhibit decomposition, making them ideal for methane synthesis (Moore and Basiliko 2006). Rain-fed bogs, dominated by woody shrubs, further support these conditions (Godin et al. 2012).

Climate change exacerbates methane emissions by altering peatland hydrology. Rising temperatures reduce soil moisture, promoting heterotrophic respiration and increasing methane formation (Salimi et al. 2021). Conversely, water saturation and anoxia enhance microbial methane production (Jiang et al. 2020). The carbon balance in peatlands is thus governed by both temperature and hydration (Keller et al. 2004, Antala et al. 2022). Microbial communities play a central role in this process, yet their precise impact on carbon cycling remains an area of active research (Preston et al. 2012, Wu et al. 2024).

Methanogenesis pathways vary: hydrogenotrophic methanogens reduce CO₂ using H₂ or formate; aceticlastic methanogens (e.g. *Methanosarcina, Methanosaeta*) convert acetate and account for ∼75% of peatland methane production; and methylotrophic methanogens utilize methylated compounds (Zinder 1993, Schulz and Conrad 1996, Metje and Frenzel 2007). Many methanogens, such as those in the phylum Crenarchaeota, remain unculturable, limiting our understanding of their ecology.

Since 2006, biogenic methane emissions have increased at three times the rate of fossil methane, underscoring the importance of microbial processes in current climate dynamics (Lan et al. 2021). While fossil methane originates from ancient organic deposits, biogenic methane is produced from recent microbial activity (Schaefer et al. 2016). Despite this, only 7% of excess atmospheric methane is removed by soil microbes, compared to 33% by atmospheric hydroxyl radicals (Jackson et al. 2020).

Anaerobic oxidation of methane (AOM) offers a promising mitigation pathway. This microbial process reduces methane emissions via redox reactions with electron acceptors such as nitrate, sulphate, iron, and CO₂, particularly in waterlogged soils (Li et al. 2023). AOM can reduce atmospheric methane by 10–60%, removing up to 0.3 × 10¹² T of CH_4_ annually (Segarra et al. 2015, Lidong et al. 2022).

Environmental factors such as moisture, organic matter, and nitrogen availability regulate microbial activity and methane production (Semenov et al. 2019, Brandt et al. 2024). Oxygen exposure accelerates organic matter degradation, while elevated CO₂ levels and nitrate concentrations can respectively enhance methane synthesis and suppress methanotrophy (van Groenigen et al. 2011, Bodelier and Frenzel 2024). Human activities, deforestation, agriculture, mining, and pollution, further disrupt peatland stability (Grzybowski and Glińska-Lewczuk 2020).

As peatlands are both a source and a sink in the global carbon cycle. Their role in methane dynamics underscores the importance of preserving these ecosystems and integrating them into broader climate policy frameworks. Continued research into microbial processes, hydrological controls, and climate interactions is vital to predict and manage the future trajectory of peatland GHG emissions.

### Future considerations for carbon monitoring

Future Earth system projections must go beyond surface-level carbon accounting and explicitly consider microbial feedback loops. Doing so will not only improve the realism of model outputs but also provide practical avenues for managing terrestrial ecosystems in a warming world. As microbial communities mediate essential functions such as decomposition, nutrient cycling, and organic matter stabilization, their integration into climate models is vital for predicting carbon fluxes under various land-use and emission scenarios. Moreover, the potential to intentionally manipulate microbial processes for climate mitigation opens a new frontier in soil management. Emerging techniques such as microbial inoculation, soil microbiome transplantation, and the promotion of high carbon-use-efficiency taxa represent promising tools to enhance soil carbon sequestration. These approaches must be informed by deep ecological understanding and supported by long-term field studies that evaluate trade-offs and system resilience. Ultimately, integrating microbial ecology into climate change science is not merely a refinement of existing models—it represents a paradigm shift. It acknowledges soils not just as passive carbon reservoirs but as dynamic, biologically active systems whose microbial constituents are both responsive to and influential upon the changing climate. Future research should prioritize this frontier through multidisciplinary partnerships, innovative technologies, and policy-relevant applications that position soil microbiomes as critical allies in climate mitigation and sustainable land stewardship.

### Nitrogen cycling within soil environments

The impacts of climate change within soil environments often focus on carbon cycling. However, climate change and anthropogenic activities can have a major impact on how nitrogen is cycled resulting in changes to the release of nitric and nitrous oxide.

The nitrogen cycle describes a series of events in which nitrogenous materials are cycled within environments transforming atmospheric nitrogen (N_2_) into simple organic forms (nitrates and nitrites) that are usable by plants before nitrogen species are converted back to atmospheric nitrogen via the stepwise process of denitrification (Jetten 2008).

### Nitrogen Fixation

Each process within the nitrogen cycle is driven by different groups of microorganisms through reduction and oxidation of nitrogen and the involvement of specific enzymes. Since atmospheric nitrogen cannot be utilized by plants directly, it is converted into ammonia via nitrogen fixing bacteria known as diazotrophs, which forms the basis of biosynthesis of organic nitrogenous compounds. Diazotrophs are commonly identified using molecular methods via the presence of “*nif* genes” within the *nif* operon responsible for production of nitrogenase reductase.

Ammonia formed from nitrogen fixation is incorporated into organic matter such as glutamine which could then be used to build nucleic acids and protein. Nitrogen fixation is limited to environments in which nitrogenase is protected from exposure to molecular oxygen (Jetten 2008), such as in the nodules of leguminous plants.

### Nitrification

In this process, NH_4_ is converted first to nitrite by a small group of nitrite oxidizing bacteria and archaea converts the nitrite produced to nitrate which is usable by plants. The gene responsible is archaeal ammonium monoxygenase (*amoA*) (Jetten 2008, Kuypers et al. 2018).

### Denitrification

Nitrates produced by the nitrification process are reduced in a step-wise manner back to N_2_ via nitric oxide (NO) and nitrous oxide (N_2_O). Many different microorganisms and enzymes are involved in this process most commonly identified via functional genes including nitrate reductase (*Nar*), nitrite reductase (*Nir*)—which is either copper containing as *NirK* or cytochrome cd1-containing as *NirS*, nitric oxide reductase (*Nor*) and nitrous oxide reductase (*Nos*) (Yang et al. 2022, Mattoo and Suman 2023).

## Challenges associated with Nitric Oxide (NO) and Nitrous Oxide (N_2_O)

Cycling of nitrogen within environments is essential. However, several environmental pollutants result from poorly controlled N cycling. Nitrates and nitrites can form nitrogen-based pollutants when formed in excess as their water-soluble nature means they can easily leach into groundwater leading to nutrient leaching and eutrophication. Additionally, and particularly when soils are subjected to increased N addition from fertilisers, atmospheric pollutants ammonia (NH_3_), nitrous oxide (N_2_O) and nitric oxide (NO) can be released. Nitrous oxide is a potent greenhouse gas (GHG) with a global warming potential 310 times higher than carbon dioxide (Aryal et al. 2022), and nitric oxide is a precursor of tropospheric ozone and contributor to acid rain (Medinets et al. 2021). Whilst these N species can be formed abiotically the majority is formed via microbial activity with environmental conditions, particularly N input, temperature and water availability key. Typically increased nitric oxide is released from dry, aerated soils, and nitrous oxide emitted from wet, anaerobic soils (Medinets et al. 2015). Atmospheric nitrous oxide has increased due to anthropogenic activities by 18% over pre-industrial levels and is expected to reach 8.316 Teragrams of N_2_O–N per year in 2050 (Aryal et al. 2022).

Changes in rainfall associated with global temperature changes can lead to the ‘Birch’ effect, where dry–wet cycles result in substantial annual emissions of NO and N_2_O from soil, as changes in water availability lead to the isolation and reestablishment of hydrological connectivity which impacts on microbial community activity, often suppressing soil enzyme activities (Medinets et al. 2021, Mattoo and Suman 2023). Intense rainstorms can lead to increased release of N with leaching reported to be up to 14 times worse than in non-disturbed areas (Gustine et al. 2022) and with prolonged rainfall and flooding leading to mobilisation of N within topsoil further exacerbating issues of eutrophication (Sinha et al. 2017). Changes in rainfall have also been shown to have significant impact on initial nitrogen fixation within soils with a recent meta-analysis by Liao et al. (2025) showing annual precipitation followed by soil organic carbon, total nitrogen and soil moisture to be the major factors affecting global free-living nitrogen fixation within soils.

Climate change can result in increased freeze-thaw events which effect labile C within the soil in turn impacting on increased catabolic and anabolic microbial activities and increasing N mineralization, nitrification and N assimilation (Mattoo and Suman 2023).

## Methods for measuring microbial change linked to biogeochemical cycles

Microbial communities within soil environments are typically densely populated with high taxonomic diversity. These communities are often unique to their environments while reflecting evolutionary patterns (Duygan and Meer 2022). Understanding their composition and dynamics is essential for elucidating ecological roles and responses to environmental changes. This requires not only quantifying key taxa and their physiological states but also considering the nature of microbial interactions, spatial distribution, and functional roles within the community (Geesink et al. 2024).

Traditionally, culture-based methods have been the cornerstone of microbial characterisation, offering opportunities for physiological and biochemical testing (Hugerth and Andersson 2017). However, these methods are limited by the need for precise knowledge of microbial nutritional requirements. Direct microscopy has revealed that microbial counts in soil far exceed colony-forming units (CFUs) obtained through culturing, with only ∼0.1% of soil microbes being culturable using standard media (Hill et al. 2000, Vieira and Nahas 2005). However, the development of approaches such as The BIOLOG system, a culture-based method, assessing microbial function by measuring the utilisation of 95 carbon sources, provides insights into community-level physiological profiles and can complement the molecular approaches discussed later (Preston-Mafham et al. 2002).

To overcome the limitations of culture-based approaches, culture-independent methods have become increasingly prevalent. These mostly include the extraction and quantification of nucleic acids from soil samples. Nucleic acid-based techniques, particularly those targeting conserved and variable regions of rRNA genes (e.g. 16S, 18S, and 5S), have proven powerful for assessing microbial diversity across all domains of life (Anahtar et al. 2016, Church et al. 2020).

The advent of high-throughput “omics” technologies has revolutionised microbiome research. Metagenomics, metatranscriptomics, metaproteomics, and metabolomics have expanded our understanding of microbial diversity and functional potential (Galloway-Peña and Hanson 2020, Kumar et al. 2024).

Many current approaches studying impacts of environmental change on microbial communities use a combined molecular approach. The measurement of environmental factors such as temperature, pH, C/N ratio and nutrient concentrations can all influence microbial activity (Wen et al. 2017), and are widely used to support microbial community data.

The overall prokaryotic community can be measured using 16S rRNA amplicon sequencing, or other longer read sequencing approaches. These techniques use nucleic acids extracted from the soil and allow fingerprints of the microbial community structure to be determined. Boyle et al., (2024), Taylor et al., (2023) and Orr et al., (2021) all show examples of this approach. Depending on the aspect of the microbial community of interest these approaches can allow functional information to be inferred by identifying the presence of microbes which are known to carry out critical roles. For example, methane emissions are governed by the balance between methanogenesis and methane oxidation, both mediated by microbial processes (Kharitonov et al. 2021). Methanogenesis is entirely carried out by methanogenic archaea and the identification of changing abundances of these microbes could indicate changes in methane release. However, it is often difficult to ascertain this information from sequence data alone particularly as some biogeochemical processes, for example nitrogen fixation have broad taxonomic range making identification via genera problematic (Dos Santos et al. 2012).

To target specific biogeochemical functions, molecular methods such as quantitative PCR which uses fluorescent probes to monitor DNA amplification in real time, allowing for accurate quantification of gene copy numbers (Fierer et al. 2005, Kleyer et al. 2017) allows targeting functional genes, such as *mcrA* for methanogens and *pmoA* for methanotrophs, have enabled accurate identification and quantification of these key microbial groups (Rivett and Bell 2018).

These gene markers have been successfully used to estimate microbial populations and identify environmental factors influencing methane cycling (Kolb et al. 2003; Kolb et al. 2009; Kharitonov et al. 2021).

Genes involved in nitrogen cycling have also been used to monitor microbes involved in biogeochemical cycling within soils with the *nirK, nirS* and *nosZ* gene frequently used (Kandeler et al. 2006, Yang et al. 2022). This information is particularly useful when combined with lab-based studies which can monitor emissions of nitrogenous based gases from the tested soils, correlating microbial data with gas release (Yang et al. 2022)

Molecular advancements have deepened our understanding of microbial diversity, interactions, and ecosystem functions. The integration of qPCR and metagenomics provides powerful tools for monitoring microbial communities in soil, offering critical insights for climate change mitigation and sustainable ecosystem management. However, nucleic acid-based measures will always be limited to the understanding of microbial genomics and transcriptomics. Whilst this gives a good overview of individual microorganisms present and the potential of the microbial community there may be differences in the translation of these genes into proteins and the functioning of microbial enzymes which makes quantification of nutrient cycling change difficult. Although more expensive and with complexities in sample-extraction, proteomics could allow a more detailed examination of soil microbial enzymes and therefore functionality linked with biogeochemical cycling (Starke et al. 2019).

Advances in the measurement of greenhouse gas emissions from soils also aids study. These approaches can either be chamber-based, where chambers are placed over small areas of soil capturing released gases which are then typically measured using gas chromatography, or via micrometeorological techniques (Zaman et al. 2021).

### Future perspectives on soil microbiome and climate change—bacteriophage

Bacteriophages, viruses that infect bacteria, play a crucial role in shaping soil ecosystems (Siyanbola et al. 2024). They are the most abundant biological entities in soil and significantly influence how nutrients cycle through the environment, which in turn affects climate change responses. Being the most abundant biological entities in soil, phages infect and break down bacteria. Through a process called “viral shunt”, they release important cellular components like DNA, proteins, and lipids back into the soil (Hampton et al. 2020, Wieczynski et al. 2023). This release boosts the availability of nutrients such as carbon, nitrogen, and phosphorus, improving soil fertility and even changing its physical structure. At the same time, phages act as powerful forces that shape bacterial communities. They can reduce harmful bacteria, which helps protect plants, but sometimes they also disrupt beneficial bacteria networks that support plant growth and health (Naureen et al. 2020, Siyanbola et al. 2024). Research shows that rising soil temperatures may shift phage lifecycles toward lytic dominance, amplifying microbial turnover and thus speeding up nutrient cycling. While this can increase soil nutrient availability, it also risks upsetting the balance of soil ecosystems if essential symbiotic microbes decline (Braga et al. 2020).

Climate change is making the interactions between phages and their bacterial hosts in soil more complex by changing factors like soil moisture, temperature, and salinity conditions that affect how microbes respond to infection (Naureen et al. 2020, Hao et al. 2025). Metagenomic studies demonstrate that during drought conditions, soil drying prompts a shift toward lysogenic infections, facilitating gene exchange and microbial resilience. Conversely, wet conditions favor lytic cycles that rapidly reshape community structure, potentially accelerating greenhouse gas (GHG) emissions by activating microbes involved in carbon and nitrogen cycling. This is significant given soil respiration contributes to major positive feedback in climate warming known as soil carbon feedback (Xu et al. 2021). Phages that target specific bacteria, such as sulfate-reducing *Desulfovibrio* species, hold promise for reducing harmful gases like hydrogen sulfide. Lab experiments using phage therapy have shown reductions in these emissions, suggesting new ways to control greenhouse gases at the microbial level (Van et al. 2024).

In agricultural settings, phages are gaining attention as climate-smart tools. Commercial products such as AgriPhage™ and XylPhi-PD illustrate their application in controlling crop pathogens in an eco-friendly way, offering precise, residue-free alternatives to chemical pesticides (Jones et al. 2007, Siyanbola et al. 2024). Beyond direct disease management, phage applications may enhance rhizosphere function: by selectively suppressing deleterious bacteria, phages may foster the proliferation of beneficial taxa (e.g. nitrogen-fixing or disease-suppressive microbes). Intriguingly, some phage–infected bacteria have been observed to elicit systemic immune responses in plants, analogous to systemic acquired resistance—a potential boost to crop resilience known as sustainable agriculture resilience (Ul Haq et al. 2024; X. Wang et al. 2024). However, responsible deployment requires careful navigation of challenges such as the horizontal transfer of antibiotic resistance genes via transduction (Watson et al. 2018), the limited host ranges of phages that restrict their broad-field application, and environmental instability affecting phage persistence. Advances in synthetic biology are helping to overcome these hurdles by creating engineered phages with broader host ranges, improved stability, or gene-editing capabilities. Combined with techniques like metagenomic analysis and AI-driven host prediction, these innovations could make phage applications more effective and reliable. Ultimately, phages may serve not just as pest-control agents but as active components in the microbial design of climate-resilient, sustainable agroecosystems.

### Opportunities for sustainable agriculture—harnessing microbial mechanisms for climate-resilient agriculture

Sustainable agriculture has transitioned from a desirable ideal to a global necessity. Faced with the intensifying challenges of climate change, soil degradation, and antimicrobial resistance, scientists and agricultural innovators are turning to microbial ecosystems for answers. Among the most promising avenues are microbial gene transfer processes and the diverse bacterial defence mechanisms evolved in response to bacteriophage predation. These naturally occurring systems, long known for their roles in microbial survival and adaptation, are now being re-evaluated as tools for improving soil health, boosting crop productivity, and reducing chemical inputs. A critical nexus of this exploration lies in understanding bacteriophage-mediated interactions, especially in the context of horizontal gene transfer (HGT) and host defence systems (Mayo-Munoz et al. 2023), which together shape microbial function and evolution within agricultural environments (Batinovic et al. 2019, Iqbal et al. 2025, Naureen et al. 2020, Siyanbola et al. 2024).

#### Horizontal gene transfer: an evolutionary force with double implications

Horizontal gene transfer is a cornerstone of microbial adaptability that allows microbes to quickly share genes, even between unrelated species (Kogay et al. 2024). In agriculture, this process helps dissemination of beneficial traits such as nitrogen fixation, stress tolerance, and degradation of xenobiotics. Genes encoding for these functions can move across microbial communities in soil, especially under the selective pressures imposed by fertilizer application, monoculture, and climate variability. However, the same mechanism also facilitates the spread of less desirable traits, including antibiotic resistance genes (ARGs) and virulence factors. However, HGT is a double-edged sword—it can also spread harmful traits, including antibiotic resistance genes (ARGs) and factors that increase bacterial virulence (Watson et al. 2018). Because of this, careful monitoring and thoughtful management of HGT in soil ecosystems are essential (Ajayi et al. 2024, Goh et al. 2024).

Bacteriophages are powerful drivers of HGT, particularly through transduction (Hampton et al. 2020). This process allows phages to transfer genetic material between host bacteria, sometimes turning beneficial or neutral strains into opportunistic pathogens. For example, the plant-associated bacterium *Pseudomonas syringae* can acquire genes for virulence via phage-mediated transfer, shifting its role from promoting plant growth to causing disease (Hulin et al. 2023, Xin et al. 2018). Such changes are not uncommon, phage genomes often carry accessory genes that can include toxins, efflux pumps, or metabolic regulators. These mobile genetic elements, when transferred in agricultural settings, can have profound effects on plant health and soil microbiome dynamics (Chao et al. 2023). Understanding these genetic exchanges is crucial for designing microbial communities that support, rather than compromise, sustainable agriculture farming practices.

#### Phage therapy and biocontrol: towards precision agriculture

Phage therapy is gaining renewed momentum as an eco-friendly alternative to synthetic pesticides and antibiotics in agriculture. Unlike broad-spectrum chemical treatments that disrupt beneficial soil microbes, bacteriophages offer specificity by targeting only their bacterial hosts while leaving the surrounding beneficial microbiota unharmed (Czajkowski et al. 2025). This precision is increasingly relevant in agriculture, where maintaining soil microbial diversity is key to long-term productivity and resilience (J. Wang et al. 2024).

Research has demonstrated that phage cocktails—combinations of different phages with overlapping host ranges can effectively target plant pathogens such as *Ralstonia solanacearum, Xanthomonas campestris*, and *Erwinia amylovora*. These treatments reduce crop losses while leaving beneficial microbes such as Actinobacteria unharmed, making them a promising tool for sustainable farming (Kering et al. 2019). Despite their promise, phage applications face several ecological and evolutionary hurdles. One major challenge is the emergence of phage-resistant bacterial mutants. Bacteria can alter or mask their phage receptors, deploy abortive infection systems, or activate more advanced immunity mechanisms such as CRISPR-Cas systems (Mayo-Munoz et al. 2023). Interestingly, many resistance mutations come at a fitness cost, such as reduced motility, biofilm formation, or virulence—traits critical for plant colonization or infection (Wang et al. 2023). This trade-off suggests that phage therapy could exert selective pressure that benefits the plant host, even in cases where complete eradication of the pathogen is not achieved. To keep pace with evolving bacteria, researchers are developing co-evolutionary strategies where phage cocktails are regularly updated or engineered alongside their bacterial targets, helping to sustain long-term effectiveness in ever-changing soil environments (Torres-Barcelo 2018).

Beyond using whole phages, researchers are exploring phage-derived proteins such as endolysins, enzymes that degrade the bacterial cell walls during the final stage of the phage infection (Nazir et al. 2023). When applied externally, especially against gram-positive pathogens, endolysins act as potent, targeted antimicrobials. For gram-negative bacteria, which possess an outer membrane that limits penetration, combining endolysins with membrane-disrupting agents like EDTA or citric acid has proven effective. These combinations can be formulated into foliar sprays or seed treatments, offering biodegradable and targeted protection against specific plant diseases (Sisson, Fagerlund et al. 2024, Sisson, Jackson et al. 2024).

The effectiveness of endolysins and other phage products is further enhanced when used in synergistic combinations. Studies have demonstrated that coupling phages with bacteriocins, low-dose antibiotics, or even nanoparticles can reduce the emergence of resistance developing and improve overall antimicrobial effects (Khan et al. 2024). While most of these approaches are still being tested in labs or greenhouses, they hold great promise for wider use in the field. Such synergistic strategies align well with integrated pest management (IPM) goals, emphasizing precision, sustainability, and environmental safety.

### Prospective of anti-phage defence in sustainable agriculture

The arms race between bacteriophages and their bacterial hosts has reached new complexity with the discovery of a vast array of anti-phage defence systems in bacteria(Hampton et al. 2020, Mayo-Munoz et al. 2023, Pinilla-Redondo et al. 2020). Traditional barriers—like Restriction–Modification (RM) systems and CRISPR–Cas adaptive immunity—continue to dominate the landscape. RM systems, which enzymatically methylate self-DNA and cleave foreign unmethylated sequences, are found in ∼90% of bacterial genomes, often with multiple subtypes coexisting in a single cell, and frequently organized into mobile “defence islands” with translocases and specificity subunits. CRISPR–Cas systems, present in roughly half of bacterial species, offer heritable, sequence-specific defence through spacer integration and Cas-mediated cleavage (types I–VI), making them a cornerstone of bacterial adaptive immunity (Dimitriu et al. 2020). More recently, abortive infection systems—such as PrrC, Shedu, Borvo, and PD-T4-7—have been identified. These systems inflict DNA or tRNA damage, halting both phage propagation and host metabolism to protect the bacterial population at the cost of individual survival (Mayo-Munoz et al. 2024, Payne et al. 2022).

Beyond these canonical systems, novel mechanisms continue to emerge, fueling a molecular arms race. For example, SspABCD–SspE identifies and nicks unmodified, invading phage DNA via phosphorothioate DNA backbone marks, representing an elegant, targeted defensive cut that differs from broad-spectrum nucleolytic cleavage (Xiong et al. 2020). Defence islands in many bacteria are now recognized as genomic hubs where these systems coexist and sometimes synergize; their organization promotes horizontal transfer and rapid dissemination of anti-phage traits. Bioinformatic tools such as PADLOC have catalogued more than 150 defence families, while phage-targeted approaches (e.g. “guilt-by-association”) continue to expand our understanding of defence system diversity. This expanding repertoire complicates phage-based interventions in agriculture but also opens opportunities for precision-guided phage engineering (Payne et al. 2022).

Phages are not simply passive players in their battle against bacterial defences—they have evolved sophisticated Anti-Defence Systems (ADSs) that allow them to bypass or disable host protective mechanisms. For example, Anti-CRISPR proteins (Acrs), often found near regulatory Aca genes, can directly block CRISPR-Cas systems by preventing DNA recognition or cleavage, sometimes by chemically modifying the Cas proteins themselves (Pinilla-Redondo et al. 2020). Other ADSs counteract bacterial restriction-modification (RM) systems by degrading restriction enzymes, protecting target DNA sequences, or chemically altering phage genomes after they are made. Additionally, some ADSs target small-molecule signalling systems—such as CBASS or Thoeris—that bacteria use to trigger abortive infection or other defence responses, effectively shutting down these alarms. To date, over 145 distinct ADSs have been identified that counter 27 different bacterial defence families, highlighting the intense and ongoing arms race between phages and their hosts (Murtazalieva et al. 2024, Payne et al. 2022, Tesson et al. 2022).

Smart phage design for agriculture stands at the nexus of exploiting ADS knowledge and tailoring phages to overcome bacterial defences. Engineered phages can carry exogenous payloads such as enzymes that break down biofilms, lactonases that disrupt bacterial communication (quorum sensing), or antimicrobial peptides, adding layers of attack beyond simply lysing bacteria. Moreover, phages carrying synthetic or naturally occurring Acr proteins can bypass bacteria armed with CRISPR defences (Pinilla-Redondo et al. 2020), improving the chances of successful infection in complex soil and plant environments. This dual approach—targeting both the bacterial cells and their defence systems—holds great promise for creating effective phage cocktails tailored to agricultural settings where resistant strains are common.

In agricultural soil ecosystems, where microbial diversity and horizontal gene transfer abound, phage application must be strategic and ecosystem aware. Phage ADS-informed cocktails may be designed to target key plant pathogens (e.g. Erwinia, Pseudomonas) by including modules that counteract restriction enzymes or abortive infection systems. Advanced phage engineering might also incorporate genetic circuits that respond to specific soil conditions or environmental triggers, including built-in safety mechanisms such as kill-switches to prevent unintended spread. However, caution is required: unintended transduction of genes—especially antibiotic-resistance determinants—by temperate or poorly designed lytic phages may accidentally transfer harmful genes, raising ecological and human-health risks. Therefore, robust biocontainment strategies, regulatory frameworks, and ecological risk assessments must accompany the deployment of designer phages in open soil systems (Farooq et al. 2022).

High-throughput metagenomic surveys across agricultural soils can map defence–anti-defence landscapes, identifying hotspots of bacterial defence. Computational pipelines can harness host–phage sequence data to predict ADS/defence synergies and flag resistance threats. Synthetic biology tools (e.g. CRISPR/Cas editing) can knock out defence systems in bacterial pathogens to sensitize them to phage attack. Ultimately, field trials in crop systems will be critical to validate these strategies, assessing their effectiveness, stability, and impacts on non-target organisms. Long-term microbiome monitoring will provide insights into ecological effects, helping ensure that phage-based interventions contribute positively to sustainable and resilient agricultural ecosystems.

### Future outlooks for understanding biogeochemical cycling within soil environments

There has always been a need to understand biogeochemical cycling within soil environments in order to understand how to optimise their productivity from an agricultural perspective, and increasingly to help mitigate for release of carbon and nitrogen-based pollutants, helping bring balance to natural cycles caused by anthropogenic actions. As the soil microbial community is complex and diverse many past studies have focused on characterising the microbial community present during times of environmental stress. This allows key members of the microbial community to be identified. However, as the microbial community is so diverse issues of functional redundancy can mean that microorganisms which are key in one environment are not present within another but with the same ecosystem parameters performed. Future studies should aim to capture the whole ecosystem within their study. This could be by widening the aspect of the microbial community studies. For example, many studies do not consider fungal, protist and viral constituents of the microbial community which may have a key impact to the overall performance of the environment. It should also see studies moving away from simple identification of the microorganisms within the soil environments to focus on their function. The integration of real time monitoring of soils to measure gas and pollutant release and tie this to microbial community structure change is key. This in turn should allow studies which look specifically at how we can then manage this change through intervention.

## Data Availability

No new data were generated or analysed in support of this research.
